# FACETS OF CLOSE, ROMANTIC, AND INTIMATE RELATIONSHIPS IN LATER LIFE

**DOI:** 10.1093/geroni/igz038.2915

**Published:** 2019-11-08

**Authors:** Karolina Kolodziejczak, Denis Gerstorf, Karen Rook

**Affiliations:** 1 Humboldt University Berlin, Berlin, Germany; 2 University of California, Irvine, Irvine, California, United States

## Abstract

Research on the role of close social relationships for physical health and well-being in later life has received increased attention over the past decades. Yet, we are still only beginning to understand potentially underlying mechanisms such as joint goals and affectionate touch. Likewise, we also know little about the relevance of particular social facets such as the role of friends and the nature of sexuality. In this symposium, we have compiled four empirical projects that showcase current and future endeavors to address some of these long-standing questions. Ungar et al. use dyadic data from older couples to examine how shared goals with the partners and positive illusions about these joint goals relate to goal progress and relationship satisfaction. Zhaoyang and Martire analyze long-term longitudinal dyadic data from older couples to examine if and how the frequency of affectionate touch between partners predicts physical health, well-being, and relationship satisfaction five years later. Fiori et al. make use of three-wave longitudinal data from a large and representative US sample to examine the unique roles that close social ties and weaker social ties have independently of one another for age-related changes in two central aspects of affective experience. Kolodziejczak et al. use time-lag data from two cohorts of adults in late midlife to capture historical changes in the perceived importance of sexuality and the evaluation of one’s sex life. Karen Rook will integrate the insights gained from these four papers, discuss their potential and limitations, and consider directions for future research.

